# Not all reflection is equal: Reflective practice, not self‐reflection, correlates with Indonesian medical students' professional identity formation

**DOI:** 10.1111/medu.70176

**Published:** 2026-01-21

**Authors:** Indah Puspasari Kiay Demak, Alexandra Androni, Adhar Arifuddin, Nur Meity, Jelle Prins, Marco Antonio de Carvalho‐Filho, Joke Fleer

**Affiliations:** ^1^ Wenckebach Institute (WIOO) ‐ Lifelong Learning, Education, and Assessment Research Network (LEARN) University Medical Center Groningen Groningen The Netherlands; ^2^ Medical Education Unit, Faculty of Medicine University of Tadulako Palu Indonesia; ^3^ Department of Epidemiology, Faculty of Public Health University of Tadulako Palu Indonesia; ^4^ Medical Education Unit, Faculty of Medicine University of Alkhairaat Palu Indonesia; ^5^ Department Health Sciences Section Health Psychology University Medical Center Groningen Groningen The Netherlands

## Abstract

**Background:**

Professional identity formation (PIF) plays a significant role in the development of medical students, with reflection expected to help learners align their personal values with the expectations of the profession. While theoretical models propose that reflection and PIF advance hand in hand, empirical studies suggest that the various types of reflection may evolve independently. In this study, we aimed to (a) investigate the levels of PIF, reflective practice and self‐reflection and insight across academic years, and (b) assess whether reflective practice and self‐reflection and insight are significant predictors of medical students' PIF.

**Methods:**

We conducted a cross‐sectional quantitative study that included 1401 medical students from four universities in Indonesia. Participants completed a demographic questionnaire along with the Professional Identity Formation (PIF) questionnaire, Reflective Practice Questionnaire (RPQ) and Self‐reflection and Insight Scale (SRIS). We used one‐way ANOVA to examine the differences of PIF, RPQ and SRIS across the academic years; Pearson correlations to examine the association between PIF, RPQ and SRIS; and regression analysis to assess the predictive value of RPQ and SRIS on PIF.

**Results:**

Both PIF (*F* = 32.221, *p* < 0.001) and reflective practice (*F* = 6.796, *p* < 0.001) increased across academic years, while self‐reflection and insight remained stable (*F* = 1.683, *p* = 0.136). Reflective practice correlated with PIF (*r* = 0.420; *p* < 0.001), while self‐reflection and insight did not (*r* = −0.017; *p* = 0.528). Reflective practice was a significant predictor of PIF in the regression analysis (*B* = 0.674, *p* < 0.001).

**Conclusion:**

Reflection on practice associates with professional identity formation in medical students, but self‐reflection and insight do not.

## Abstract 2

Is all reflection equal? FInd out how reflective practice and self‐reflection support medical student's professional #identity formation. #MedEd

## 1 INTRODUCTION

Professional identity formation (PIF) is fundamental to the development of medical students as they become future healthcare professionals.^1^, ^2^ As students embark on their educational trajectory, their identity formation is modulated by a range of socialization experiences, particularly through participation in clinical exposures and role modelling.^3^, ^4^, ^5^ Reflecting on these complex experiences is essential, as it enables students to critically align their personal beliefs and values with the profession's expectations.^1^, ^6^, ^7^ Yet most existing literature treats reflection as a single, undifferentiated activity, and we still have limited quantitative examinations of how different types of reflection associate with PIF.^8^, ^9^, ^10^, ^11^, ^12^, ^13^, ^14^ By identifying which types of reflection are associated (or not) with PIF, educators can design and optimize activities that more effectively support students' professional identity development.

PIF is a central developmental process in medical education through which students internalize the values, norms and roles of the profession.^1^, ^2^ Drawing on Wenger's community of practice theory, PIF can be understood as a trajectory of becoming a member of the medical community; a trajectory that is nurtured by participation in shared practices and recognition as a legitimate member of that community.^15^, ^16^ Clinical and educational settings function as spaces where this participation happens, where students encounter both formal and informal influences and continually interpret and negotiate the values related to the process of becoming a doctor.^17^, ^18^ Because becoming a doctor is an interpretive process, students need opportunities to make sense of their experiences and reconcile emerging professional expectations with their own values.^1^, ^16^, ^19^, ^20^ In this regard, reflection provides a key mechanism for examining, negotiating and articulating developing identities.^7^, ^9^, ^13^, ^21^

Within medical education, two types of reflection are especially relevant. The first one is reflective practice, first articulated by Schön,^22^ which refers to the deliberate, critical analysis of one's experiences aimed at improving future clinical practice, decision‐making and patient care. Reflective practice relies fundamentally on the development of reflective capacity, which encompasses a learner's ability, motivation and tendency to engage in thoughtful consideration of their experiences throughout both academic and clinical contexts.^23^, ^24^ This process involves critically examining one's own and others' assumptions and beliefs about clinical decisions, attending to emotions (e.g., empathy, discomfort and moral distress) as sources of insight and remaining responsive to the interpersonal and contextual dynamics of clinical settings, while maintaining an open‐minded stance toward learning from diverse perspectives.^22^, ^25^, ^26^, ^27^ Engaging in reflective practice supports the development of critical thinking skills, fosters cognitive empathy and cultivates not only self‐awareness but also a deeper understanding of patients' perspectives; however, its direct contribution to PIF has not yet been empirically demonstrated.^28^, ^29^

The second form, self‐reflection and insight, focuses on the ability to critically evaluate one's thoughts, feelings and behaviours.^30^ The concept of self‐reflection is grounded in self‐regulation theory, which emphasizes how individuals monitor and adjust their behaviours to achieve personal goals.^31^ In this view, self‐reflection represents a metacognitive stance toward understanding oneself rather than the accuracy of performance judgements.^32^ Building on this framework, Grant et al.^30^ proposed a generic model of self‐regulation and goal attainment that highlights the complementary roles of self‐reflection and insight. While self‐reflection pertains to evaluating thinking about one's internal process, insight refers to the deeper understanding of those internal experiences, including emotions, thoughts and behaviours.^30^ In the context of medical education, self‐reflection supports learners track their progress, self‐regulate and adjust their behaviour toward professional goals.^33^ However, empirical studies have primarily linked self‐reflection to perceptions of professionalism, competence and empathy—rather than directly to PIF.^34^, ^35^

Despite the recognized theoretical connection between reflection and PIF, the existing literature presents several limitations. While a number of qualitative studies have explored the relationship between reflection and PIF,^8^, ^9^, ^10^, ^21^, ^36^ there is a notable lack of quantitative evidence confirming this link. Quantitative investigations, where available, have not yet demonstrated a direct relationship between reflective practice and self‐reflection and the development of PIF in medical students.^37^, ^38^

Adding to the above, prior research in medical education demonstrates that PIF generally strengthens as students advance through medical school.^38^ However, findings on students' reflection show a more fragmented picture: some studies report increases in reflection scores, while others observe decreases or irregular patterns across the curriculum.^39^, ^40^, ^41^, ^42^ Although theoretical models propose that PIF and reflection should evolve synchronously,^1^ this relationship is not consistently supported. Such inconsistencies suggest that the relationship between PIF and reflection may not be straightforward or linear, highlighting the need for a more nuanced understanding of how these constructs interact across different academic years.

To address these gaps and advance our understanding of how reflection supports PIF, the present study examined two interrelated forms of reflection—reflective practice and self‐reflection and insight—concurrently within a single model. By exploring their contributions to PIF across different stages of medical education, this study provides a more comprehensive and nuanced perspective than previous work. Specifically, we aimed to (a) assess the levels of reflective practice, self‐reflection and insight and PIF among medical students across academic years and (b) determine whether these forms of reflection significantly predict students' PIF.

## 2 METHODS

### 2.1 Study design

We conducted an institution‐based quantitative cross‐sectional study to investigate the influences of reflective practice and self‐reflection and insight on medical students' PIF. This study was a part of a multisite umbrella study executed in November and December 2024 to students at four medical schools in Indonesia.

#### 2.1.1 Context

Medical education in Indonesia is structured as a 6‐year undergraduate curriculum, conducted in Bahasa Indonesia and divided into two phases: the pre‐clinical (Bachelor) phase and the clinical (Medical degree) phase. The pre‐clinical phase lasts 3.5 to 4 years (seven or eight semesters depending on the university curriculum). During this phase, students engage in both campus‐based academic activities and community‐based learning activities designed to build foundational medical knowledge and skills. Following successful completion of the pre‐clinical phase, students' progress to the clinical programme, which spans 2 years and constitutes the professional training phase. During the clinical programme, students are trained in hospitals or public health centres, where they interact directly with patients. In this setting, students perform medical history taking (anamneses), physical examinations and propose diagnoses and treatment plans for the patients they care for under the supervision of their instructors. Once completing this phase, students are awarded their medical doctor certificate.

### 2.2 Participants and procedure

We employed total sampling and invited all medical students to participate by asking school administrators at the respective institutions to distribute a link to an online survey through official WhatsApp group chats. These group chats, organized by academic year, included all students and were strictly used by administrators to share academic schedules and formal faculty announcements. We also placed posters in several locations on campus and in hospitals affiliated with the clinical phases of the curriculum. The survey was accessible via the Qualtrics digital platform and allowed participants to complete it over multiple sessions. If students did not complete the questionnaire in one session, they could return using the same link and device to continue answering the remaining questions. Participants had up to 1 month to complete the survey. Those who completed the survey received a mobile phone top‐up compensation worth IDR 30000 (≈ USD 2) for their time and participation.

Ethical approval for this study was obtained from the Medical and Health Research Ethics Committee of Tadulako University (No. 460/UN28.1.30/KL/2024). Participants were asked to provide informed consent before proceeding with the survey. All data were collected anonymously and treated confidentially.

### 2.3 Measurement instruments

The primary survey consisted of seven questionnaires, administered in a randomized order to control for potential sequence effects.^43^ Each questionnaire corresponded to one of the key variables we sought to study, namely, PIF, reflective practice and self‐reflection and insight. For the PIF questionnaire, a validated Bahasa version was available. However, no validated Bahasa versions existed for the questionnaires to measure reflective practice and self‐reflection and insight. We therefore translated these scales into Bahasa following the regular translation‐backtranslation procedure^44^, ^45^ and performed an internal validation for these two questionnaires. The methods and results of the psychometric analyses are described in detail in the Supporting Information.

#### 2.3.1 PIF

PIF was measured using the PIF questionnaire developed by Susani and colleagues^38^, ^46^ for Indonesian medical students. We selected this scale because its items capture relational and collective aspects of professional identity, which align with dominant collectivist values in the Indonesian context. It consists of 17 items distributed across three subscales: (1) perceived comfort in the medical professional community (five items), (2) enthusiasm to engage (five items) and (3) effectiveness in fulfilling a professional role (seven items). Responses are measured on a 7‐point Likert scale, ranging from 1 (*very unlikely*) to 7 (*very likely*). An additional ‘not applicable’ option—scored 0—is provided for participants who have not encountered the described experience. Cronbach's *α* of the PIF scale was 0.80 (Supporting Information).

#### 2.3.2 Reflective practice

Reflective practice was measured using the Reflective Practice Questionnaire (RPQ), which was initially developed by Priddis and Rogers.^23^ We only included the reflective capacity subscales that have been used in medical education, which are Reflection‐in‐action, Reflection‐on‐action, Reflection with others and Self‐appraisal.^27^ Each subscale has four items, totalling 16 items. For the response format, we used the updated version of the RPQ,^26^ which employs a 6‐point Likert scale that we judged to be easier for students to understand: (1) *Very rarely*, (2) *Rarely*, (3) *Sometimes*, (4) *Often*, (5) *Very often*, (6) *Almost always*. Our psychometric analyses demonstrated acceptable internal consistency for the subscales (Cronbach's *α* = 0.87–0.90), but revealed high inter‐factor correlations between the subscales (*r* = 0.85–0.91), suggesting limited discriminant validity and supporting a unidimensional structure. Consequently, we treated the instrument as a single scale measuring student engagement in reflective practice. The total scale showed excellent internal consistency, with Cronbach's *α* = 0.96 (Supporting Information).

#### 2.3.3 Self‐reflection and insight

Self‐reflection and insight were assessed using the Self‐Reflection and Insight Scale (SRIS), developed by Grant et al^30^ and first applied to medical students by Roberts and Stark.^33^ The original scale consists of 20 items across three subscales: Engagement in self‐reflection, Need for self‐reflection (both representing the self‐reflection domain) and Insight. Each of the two self‐reflection subscales contains six items, while the Insight subscale includes eight items. Responses are rated on a 5‐point Likert scale ranging from 1 (*strongly disagree*) to 5 (*strongly agree*). Our psychometric analyses confirmed the three‐factor structure of the instrument but suggested the removal of seven items to improve model fit. After inspection of these items, we decided to remove them and conducted another confirmatory factor analysis. The revised scale with the remaining 13 items demonstrated acceptable internal consistency, with Cronbach's *α* = 0.66 for *Engage in reflection* (three items), 0.79 for *Need for reflection* (five items) and 0.86 for *Insight* (five items) (Supporting Information).

### 2.4 Statistical analyses

We performed descriptive statistical analyses to profile the demographic characteristics of our participants. Means and standard deviations (SD) were calculated for continuous variables and total scores per instrument. Since reversed items in the SRIS were pre‐coded within the Qualtrics platform, we retrieved data that already reflected the reversed scoring.

We performed one‐way ANOVA to examine differences in mean scores of PIF, reflective practice and self‐reflection and insight across academic years and independent *t*‐test to assess differences in gender and programme. Significant results were followed by Bonferroni post hoc tests and calculations of Cohen's *d* to determine the magnitude of differences. Effect sizes were interpreted based on Cohen's^47^ criteria: 0.01–0.19 as very small, 0.20–0.49 as small, 0.50–0.79 as medium and ≥80 as large.

Correlation analyses were conducted to assess the association between PIF, reflective capacity and self‐reflection and insight. Predictor variables that demonstrated statistically significant correlations with PIF were subsequently entered into a multivariable linear regression model to estimate their independent associations with PIF.

We analysed the data at the student level because, although students were nested within four medical schools, the participating schools followed a similar curriculum and collected data in the same time frame. Moreover, one‐way ANOVAs indicated that between‐school effect sizes were negligible for all key variables (see Supporting Information).

All analyses were performed using IBM SPSS Statistics v29.0 and R Studio v12.0, with a significance level set at 0.05. To address the small amount of randomly missing data, we applied SPSS's automatic replacement function, which imputes values based on the observed distribution. Although this is a single imputation and may lead to some underestimation of variance, it allowed us to retain cases for subsequent analyses and to minimize potential loss of statistical power.

## 3 RESULTS

### 3.1 Sample description

Of 3649 medical students invited to participate, 2474 responded to the survey, yielding a response rate of 67.8%. After excluding participants who declined to participate, submitted duplicate entries or had missing relevant data (i.e., incomplete PIF questionnaire, RPQ or SRIS), a total of 1401 were included in the final analysis, making the usable response rate 39%. Approximately 65% of respondents were pre‐clinical and 70% female (Table 1). This higher proportion broadly reflects the structure of enrolment at the study site, where pre‐clinical cohorts are numerically larger due to the longer duration of the pre‐clinical phase, and recent intakes have been female‐dominant.

**TABLE 1:** Participants' characteristics.

Characteristics	Total (*n* = 1401)
Age	M 20.98 (SD 2.16)
Gender	
Male	412 (29.4%)
Female	989 (70.6%)
Programme	
Pre‐clinical	905 (64.6%)
Clinical	496 (35.4%)
Academic year^a^	
Year 1 pre‐clinical	234 (16.7%)
Year 2 pre‐clinical	162 (11.6%)
Year 3 pre‐clinical	307 (21.9%)
Year 4 pre‐clinical	202 (14.4%)
Year 1 clinical	257 (18.3%)
Year 2 clinical	239 (17.1%)

^a^ Participants with delayed graduation were assigned to the final year of their current phase (e.g., a fifth‐year pre‐clinical student was categorized as a fourth‐year student).

### 3.2 Differences across academic years

To address our first aim—examining the levels of PIF, reflective practice and self‐reflection and insight across academic years—we plotted line graphs of the mean score for each construct from Year 1 pre‐clinical through Year 2 clinical, performed comparative analysis and continued with post hoc analyses to explore significant differences.

#### 3.2.1 PIF

**FIGURE 1:**
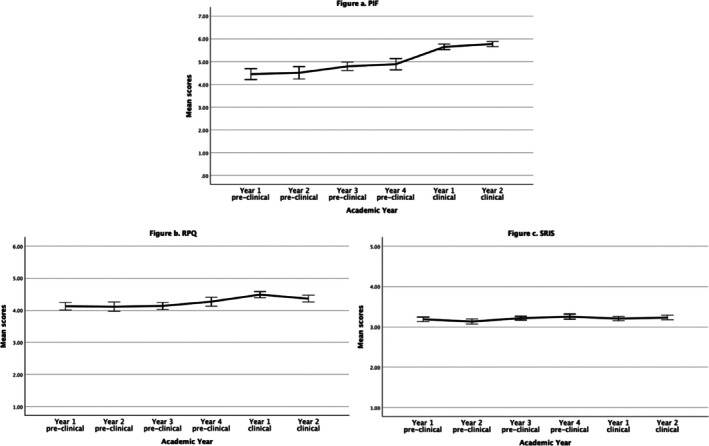
Line graph of the mean scores of PIF (a), RPQ (b) and SRIS (c) across academic years.

The trend of increasing scores indicated a slight steady rise in PIF from the first to the fourth pre‐clinical year, followed by a more substantial increase at the transition from pre‐clinical to clinical year (Figure 1a). The comparative analysis showed a significant difference in PIF scores (*F* = 32.221, *p* < 0.001) (Table 2). Following the omnibus ANOVA, we performed Bonferroni post hoc tests to identify pairwise differences in PIF scores across academic years. Students in the first pre‐clinical year scored significantly lower than those in the fourth pre‐clinical year (mean difference = −0.44; *p* = 0.043) (Table 3). Across the cohort, all pre‐clinical years scored lower than the clinical years ranging from −0.76 to −1.33 (*p* < 0.001).

**TABLE 2:** Comparative analysis of gender, programme and academic year on PIF, RPQ and SRIS.

Variables		PIF					RPQ					SRIS				
		Mean	SD	*F*	Sig.	Cohen's *d*	Mean	SD	*F*	Sig.	Cohen's *d*	Mean	SD	*F*	Sig.	Cohen's *d*
Gender	Male	5.10	1.53	N/A	0.428	0.05	4.32	0.88	N/A	0.092	0.1	3.16	0.45	N/A	0.013	0.15
	Female	5.02	1.53				4.23	0.94				3.23	0.43			
Programme	Pre‐clinical	4.68	1.77	N/A	<0.001	0.68	4.16	0.97	N/A	<0.001	0.29	3.20	0.43	N/A	0.517	0.04
	Clinical	5.71	0.96				4.43	0.8				3.22	0.45			
Academic year	Year 1 pre‐clinical	4.45	1.87	32.221	<0.001	N/A	4.13	0.93	6.796	<0.001	N/A	3.19	0.43	1.683	0.136	N/A
	Year 2 pre‐clinical	4.51	1.77				4.11	0.94				3.13	0.39			
	Year 3 pre‐clinical	4.80	1.65				4.14	0.99				3.22	0.41			
	Year 4 pre‐clinical	4.89	1.79				4.27	1.01				3.25	0.45			
	Year 1 clinical	5.65	1.00				4.49	0.77				3.21	0.45			
	Year 2 clinical	5.78	0.90				4.36	0.82				3.23	0.45			

Abbreviation: N/A, not applicable.

**TABLE 3:** Post hoc test of academic years.

Academic year		PIF		RPQ	
		Mean difference	Sig.	Mean difference	Sig.
Year 1 pre‐clinical	Year 2 preclinic	−0.06	1.000	0.01	1.000
	Year 3 pre‐clinical	−0.34	0.147	−0.01	1.000
	Year 4 pre‐clinical	−0.44*	0.043	−0.14	1.000
	Year 1 clinical	−1.20*	<0.001	−0.36*	<0.001
	Year 2 clinical	−1.33*	<0.001	−0.24	0.075
Year 2 pre‐clinical	Year 3 pre‐clinical	−0.28	0.851	−0.02	1.000
	Year 4 pre‐clinical	−0.38	0.286	−0.16	1.000
	Year 1 clinical	−1.14*	<0.001	−0.38*	<0.001
	Year 2 clinical	−1.27*	<0.001	−0.25	0.107
Year 3 pre‐clinical	Year 4 pre‐clinical	−0.09	1.000	−0.13	1.000
	Year 1 clinical	−0.86*	<0.001	−0.35*	<0.001
	Year 2 clinical	−0.98*	<0.001	−0.23	0.062
Year 4 pre‐clinical	Year 1 clinical	−0.76*	<0.001	−0.22	0.162
	Year 2 clinical	−0.89*	<0.001	−0.09	1.000
Year 1 clinical	Year 2 clinical	−0.13	1.000	0.13	1.000

* Significant at *p* < 0.05.

#### 3.2.2 Reflective practice

RPQ scores remained relatively stable during pre‐clinical years 1–3, then increased in late pre‐clinical year 4 and continued to rise into the clinical year 1, followed by a slight decrease in clinical year 2 (Figure 1b). The one‐way ANOVA revealed significant differences in RPQ between years (*F* = 6.796, *p* < 0.001). Post hoc analysis showed that scores in the first, second and third pre‐clinical years were significantly lower than those in the first clinical year, ranging from −0.35 to −0.38 (*p* < 0.001).

#### 3.2.3 Self‐reflection and insight

For the SRIS, results revealed fluctuations with minor increases and decreases, ranging between 0.02 and 0.06 points (Figure 1c). One‐way ANOVA showed no significant difference in SRIS scores between year groups (*F* = 1.683, *p* = 0.136).

### 3.3 Predictors of PIF

To address our second aim—testing whether demographic data, reflective practice and self‐reflection and insight predict PIF—we first carried out group comparisons for gender, programme and academic year and bivariate correlation among age, PIF, RPQ and SRIS. Variables that showed significant between‐group differences or significant correlation with PIF were subsequently entered into a multivariable linear regression model.

The results showed that female students had higher SRIS scores than male, although the effect size was very small (*p* = 0.013; *d* = 0.15) (Table 2). Students in the clinical programme scored higher on PIF with a medium effect (*p* = <0.001; *d* = 0.68) and on RPQ with a small effect (*p* = <0.001; *d* = 0.29) than students in the pre‐clinical programme. Age was weakly correlated with PIF (*r* = 0.276; *p* < 0.001), whereas RPQ moderately correlated with PIF (*r* = 0.420; *p* < 0.001). In contrast, SRIS had no significant correlation with PIF (*r* = −0.017; *p* = 0.528).

The multivariable regression model accounted for 25% of the variance in PIF (*R* ^2^ = 0.25; *p* < 0.001). RPQ was the strongest predictor (*B* = 0.67, *p* < 0.001), followed by programme (*B* = 0.42, *p* < 0.001) and age (*B* = 0.08, *p* = 0.003) (Table 4). Controlling for the other covariates, PIF rises by 0.67 points for every additional RPQ point, by 0.42 points for clinical (vs. pre‐clinical) programme and by 0.08 points per year of age.

**TABLE 4:** Multivariable regression of demographic data and RPQ on PIF.

Predictors	*B*	SE	*p*	95% CI
(Constant)	−0.35	0.53	0.509	[−1.38, 0.69]
Age	0.08	0.03	0.003	[0.03, 0.14]
Programme	0.42	0.14	0.003	[0.14, 0.70]
Academic year	0.06	0.05	0.190	[−0.03, 0.16]
RPQ	0.67	0.04	0.000	[0.59, 0.75]

## 4 DISCUSSION

Our study sheds light on how reflection contributes to medical students' PIF across their medical training. To the best of our knowledge, this is the first study to investigate the association between reflective practice, self‐reflection and insight and PIF in medical education. We found that both reflective practice and PIF increased significantly over academic years, while self‐reflection and insight showed no significant change. Multivariate regression further indicated that, besides age and phase of the programme, reflective practice was an independent predictor of PIF, while self‐reflection and insight did not demonstrate a significant association with PIF. These findings suggest that forming one's professional identity is not just about acquiring knowledge but also about growing into the role through authentic patient care and structured opportunities to make sense of that care.^1^, ^10^, ^48^ In the rest of the discussion, we comment on how students develop a professional identity, and why reflective practice plays a vital role in this process.

In our cohort, we found that PIF increased progressively across the academic years. This pattern is consistent with cross‐sectional evidence from Findyartini et al.^49^ and Susani et al.,^38^ both of whom reported higher PIF scores in more advanced students. Longitudinal work by Tagawa^50^ similarly documented steady growth in professional identity over the course of students' medical education trajectories. Collectively, these findings align with the framework proposed by Cruess et al.,^1^ which posits that although PIF is inherently non‐linear and dynamic, it nevertheless tends to consolidate as students accrue time and professional experience.

We found that reflective practice was positively associated with PIF. Our findings further suggest that ‘professional identity is learned’^48^, ^51^, ^52^ and that reflection on the practice may spark a transformative process that shapes how students internalize the values, roles and responsibilities of the medical profession. This interpretation aligns with the framework proposed by Cruess et al.,^1^ who conceptualize PIF as a developmental pathway in which guided reflection enables learners to extract meaning from clinical encounters and progressively internalize the profession's core values and beliefs. Empirical evidence supports our findings, demonstrating that structured reflection and narrative exercises help novice medical students articulate emerging professional selves,^12^ and when reflective writing is purposefully scaffolded, it integrates personal experience with professional expectations.^10^ Moreover, the development of reflective skills among medical students can significantly contribute to the shaping of their professional identity.^9^

Students' PIF appears to be sparked particularly by reflective practice. Unlike general reflection tools, the RPQ used in our study roots students' reflections in the patient‐provider relationship. Anchoring learning in meaningful encounters makes the reflection process more authentic and applicable. Reflective practice, as operationalized by the RPQ, is inherently transactional and social, occurring through participation in shared professional activities (e.g., ward rounds, case discussions and debriefings) and in dialogue with mentors, peers and patients. Drawing on Wenger's concept of community of practice, we understand such reflection as occurring within a social space in which meaning and identity are co‐constructed.^16^

Our study revealed that students in their later years (clinical programme)—those most deeply embedded in clinical settings—scored significantly higher on the RPQ than those in the pre‐clinical programme. It is likely because they had more opportunities to witness the complexity and emotional labour of real‐world healthcare. As in Gishen and Chakrabarti's narratives,^53^ students recognized the importance of reflection after challenging clinical encounters, and service‐learning studies from Rogers and Van Winkle^54^ similarly show that active community engagement confronts students with real‐world dissonance that triggers reflection and perspective taking.

In contrast to reflective practice, we found that self‐reflection and insight scores remained constant across academic years. Our results align with those of Roberts and Stark,^33^ who suggest that while students may reflect on their clinical experiences, they are not necessarily taught to reflect on themselves. The lack of association between self‐reflection and insight and PIF in our study underscores this point. Developing a professional identity involves not just thinking deeply about oneself but also integrating that evolving self with external expectations and professional values. This integration can be difficult. As Buck et al.^55^ observed, students may adopt physician‐like behaviours without fully merging their personal and professional identities. Our results align with this phenomenon: students may behave ‘like physicians’ while still psychologically compartmentalizing their professional role.

Moreover, considering the centrality of the ‘being’ dimension at the heart of PIF,^51^, ^56^ our findings add nuance to this ongoing discussion. Jarvis‐Selinger and colleagues^56^ argue that the development of ‘being’ emerges from both intrapersonal (‘me‐to‐me’) and interpersonal (‘me‐to‐social’) reflection. Yet, the SRIS we used was developed as a trait‐oriented instrument intended to measure one's *need* and *tendency* to think about personal thoughts and feelings rather than reflecting through dialogue with others.^30^ Consequently, it captures only the intrapersonal component of reflection. Moreover, as a trait measure, the SRIS also lacks sensitivity to change over time and thus cannot effectively track developmental progress.^57^ As a result, SRIS scores may fail to represent the integration between personal and professional identities that occurs through social interaction.

Lastly, our findings need to be interpreted in light of cultural context. The study was conducted in Indonesia, which is often characterized as having a collectivist orientation, where PIF may be experienced less as an individual career trajectory and more as the assumption of responsibilities within a professional community that serves patients and society.^18^, ^49^, ^58^ In such contexts, conformity and social harmony are highly valued, which may discourage self‐reflection.^59^, ^60^ Moreover, the PIF scale we used emphasized alignment with professional norms, belonging to the profession and commitment to patients and teams, which resonates with these relational and collective understandings of what it means to be a physician.^38^ By contrast, more recent discussions of PIF in medical education have increasingly drawn on developmental theories.^10^, ^61^ It is therefore plausible that our PIF measure was less sensitive to individual differences in self‐reflection than a PIF measure derived from a developmental framework would have been.

### 4.1 Practical implications

Our study suggests that providing students with opportunities to engage in reflective practice may be an important pedagogical approach for supporting the development of their professional identity. To better support students in internalizing the values of the medical profession, we recommend faculty to design a curriculum with a clear, intentional sequence of reflective‐practice activities, supported by skilled mentors and protected time. Without these elements, the reflective skills critical for ongoing professional growth will not fully develop.^54^ Likewise, reflective practice should be clear and structured. Students should be aware of what happens before, during and after patient encounters, and reflection sessions ought to be led by clinical educators who are available and prepared to provide constructive feedback. Embedding these activities into routine clinical workflow is key. Reflection should be perceived as an integral component of professional practice rather than an optional task.

Regarding self‐reflection, structured written and narrative exercises like guided journaling, critical incident storytelling and facilitated learner portfolios may provide concrete scaffolds that help students align emerging professional commitments with their pre‐existing personal identities.^13^, ^21^ By cultivating an intentional, disciplined habit of self‐reflection, students can negotiate the tension between socialization (learning how things are done) and subjectification (choosing who they wish to become).^62^ Educators may consider introducing such reflective exercises early in training and revisiting them at key transition points (e.g., semester changes or entry into clinical programme). A critical element of the above is that such work must be supported by trained faculty mentors, distinct from evaluators or assessors, who can provide robust formative feedback^13^, ^63^ rather than judgement.

### 4.2 Strengths and limitations

Our study's multi‐institutional design and large sample size strengthen the robustness to the findings. Nevertheless, because we included only four schools out of 117 medical schools in Indonesia, the findings should not be assumed to be representative of all Indonesian medical schools. In addition, given the sample's pre‐clinical–clinical distribution, our inferences about increases during the clinical phase should be interpreted with caution and are best generalized to curricula with a similar structure. Another limitation is although our cross‐sectional comparison across academic years provides a preliminary overview of stage‐specific differences in reflective practice, self‐reflection and insight and PIF, longitudinal follow‐up is required to confirm within‐person developmental trajectories over time. Moreover, there is a reasonable likelihood of social desirability bias, whereby participants might have shaped their responses to meet perceived faculty expectations. This effect may be particularly pronounced in collectivist cultures, where belonging to the group is deeply valued and being excluded carries a strong and distressing psychological load.^64^ Although participation was voluntary and fully anonymous, and participants were assured that their responses would have no bearing on academic evaluations, we could not guarantee complete mitigation of this effect. Furthermore, the considerable length of the questionnaire we used may have led to respondent fatigue, as evidenced by the high response rate but relatively low completion rate. The low usable response rate introduces the possibility that respondents differed from non‐respondents, which may limit generalizability beyond students who chose to participate. To maximize engagement and improve response rates, participants were allowed to pause and resume the survey at will within a 1‐month window, and modest incentives were offered for full completion. Lastly, we analysed all responses in a single set without disaggregating by institution. Although clustering effects were negligible from a statistical perspective, students are embedded in distinct institutional cultures and hidden curricula; therefore, our results should be contextualized before extrapolating to medical schools with substantially different educational environments.

### 4.3 Future research

Studying how medical students engage in self‐reflection across their training may shed light on the processes by which personal and professional identities are integrated and how this evolves over time. Such insights can guide the design and evaluation of interventions that deliberately cultivate self‐reflection and monitor its development. We also propose the development of an instrument that captures both the intrapersonal and interpersonal dimensions of self‐reflection.

## 5 CONCLUSION

Reflective practice and PIF increased significantly across academic years, while self‐reflection and insight showed no significant change. Purposeful, context‐sensitive reflective practice was associated with PIF in medical students, whereas intrapersonal self‐reflection alone showed no significant association. Although our data did not demonstrate a statistical association between self‐reflection and PIF, we believe that self‐reflection is crucial for making meaning of experiences to themselves and integrating the personal and professional selves, thus indirectly supports PIF. In light of these associations, faculty may consider providing psychologically safe, structured opportunities for reflection early in the curriculum and continuing to scaffold such experiences throughout training.

## AUTHOR CONTRIBUTIONS

Indah Puspasari Kiay Demak, Joke Fleer, Marco Antonio de Carvalho‐Filho and Jelle Prins contributed to the study design and participated in data interpretation. Data collection was carried out by Indah Puspasari Kiay Demak and Nur Meity. Data analysis was performed by Indah Puspasari Kiay Demak and Adhar Arifuddin. Indah Puspasari Kiay Demak and Alexandra Androni were responsible for editing and reviewing the manuscript. All authors contributed to the writing of the manuscript and approved the final version.

## CONFLICT OF INTEREST STATEMENT

The authors declare that there is no conflict of interest.

## ETHICS STATEMENT

Ethical approval for this study was obtained from the Medical and Health Research Ethics Committee of the Faculty of Medicine at the University of Tadulako (No. 460/UN28.1.30/KL/2024). Participants were asked to provide informed consent before proceeding with the survey. All data were collected anonymously and treated confidentially. Participants were free to withdraw from the study at any time and for any reason, without consequences.

## Supporting information


**Table S1.** 16‐item reflective practice questionnaire (RPQ).
**Figure S1.** Reflective practice questionnaire (RPQ) with standardized path coefficient.
**Table S2.** Goodness‐of‐fit RPQ.
**Table S3.** Item response statistics of the 20‐item self‐reflection and insight scale (SRIS).
**Figure S2.** Self‐reflection and insight scale (SRIS) with standardized path coefficient of the model fit.
**Table S4.** Goodness‐of‐fit SRIS.
**Table S5.** Correlation of original and revised model of SRIS.
**Table S6.** Comparison of main variables between school and cohorts.

## Data Availability

The data that support the findings of this study are available from the corresponding author upon reasonable request.
