# Supplementary material for: Not all reflection is equal: Reflective practice, not self‐reflection, correlates with Indonesian medical students' professional identity formation
Source: Med Educ. 2026 Jan 21;60(9):976–86. doi: 10.1111/medu.70176 (PMC13441198; doi:10.1111/medu.70176)
Supplement: Supplementary file 1 [file MEDU-60-976-s001.docx]

**Supplementary Material**

1. **Reflective practice questionnaire (RPQ) and Self-reflection and insight scale (SRIS)**

Given that the reflective practice questionnaire (RPQ) and self-reflection and insight scale (SRIS) reflection instruments had not previously been validated in the Bahasa Indonesia version. Therefore, we first translated and assessed the internal validity and reliability of the instruments.

1.1 **Translation procedure**

The scales were translated into Bahasa following the regular translation-backtranslation procedure.^1, 2^ In the first step, three independent professional translators (one of whom is an expert in medical education) translated the original questionnaires into Bahasa Indonesia. The three translators discussed the translation to reach consensus, and the agreed-upon translation was then back-translated into English by a fourth independent professional translator fluent in both languages. The back translation version was comparable to the original one. After agreeing on the Bahasa Indonesia version, the RPQ and SRIS were piloted on 30 clinical students. Once the students completed the questionnaires, the first author debriefed the questionnaire items with the students. All students agreed on the clarity of the items and did not identify any statements that could have multiple interpretations.

1.2 **Psychometric analysis**

We performed confirmatory factor analysis (CFA) and reliability analysis to assess the psychometric properties of the translated instruments. CFA was used to confirm construct validity, and several goodness-of-fit indices were calculated to evaluate whether the instrument met expected values. The Root Mean Square Error of Approximation (RMSEA) was considered adequate with values < 0.08 (MacCallum et al., 1996), while the Standardized Root Mean Square Residual (SRMR) was deemed acceptable at < 0.08.^4^ A Comparative Fit Index (CFI) > 0.9 and the normed chi-square (CMIN/df) was expected to fall within the range of 1–3 was deemed acceptable at < 0.08.^4^ A Comparative Fit Index (CFI) > 0.9.^4^ Reliability testing was conducted with internal consistency assessed using Cronbach’s alpha, where α > 0.7 was considered sufficient.^5^ Data analysis were performed using R Studio v12.0, with a significance level set at 0.05.

**1.2.1 RPQ psychometric analysis results**

We applied the translated version RPQ to a sample of 1,524 participants, including 445 male and 1,079 female students, with a mean age of 20.98 years (SD = 2.16).

The CFA result of the original model showed that all indicators have significant estimates (p < 0.001) with a loading factor of more than 0.70 (Figure 1). There were strong and significant correlations among all latent variables in the model, with correlation coefficients ranging from 0.847 to 0.913. These high inter-factor correlations suggest that these subscales are interrelated, suggesting that it assesses only a single dimension. The revised model demonstrated good performance across nearly all fit indices (CFI = 0.936, TLI = 0.921, SRMS = 0.041), providing evidence of sound internal validity and justifying its use in subsequent analyses (Table 2). The reliability of RPQ was 0.96 (Table 1).

Table 1. 16-item reflective practice questionnaire (RPQ)

| **Item** | **Mean** | **SD** | **Cronbach’s α** | **CITC** | **AIID** |
| --- | --- | --- | --- | --- | --- |
| **Reflection in Action** | **4.13** | **1.01** | **0.90** |  | |
| 9.     During interactions with patients, I consider how their personal thoughts and feelings are influencing the interaction | 4.11 | 1.14 |  | 0.764 | 0.882 |
| 9. Selama berinteraksi dengan para pasien, saya mempertimbangkan bagaimana pikiran dan perasaan mereka memengaruhi interaksi tersebut. |  |  |  |  |  |
| 11.  During interactions with patients, I consider how my personal thoughts and feelings are influencing the interaction. | 4.24 | 1.15 |  | 0.738 | 0.891 |
| 11. selama berinteraksi dengan pasien, saya mempertimbangkan bagaimana pikiran dan perasaan pribadi saya memengaruhi interaksi tersebut |  |  |  |  |  |
| 15.  During interactions with patients, I recognize when my pre-existing beliefs are influencing the interaction | 4.10 | 1.14 |  | 0.827 | 0.859 |
| 15. Selama berinteraksi dengan pasien, saya mengetahui ketika keyakinan saya yang sudah ada sebelumnya memengaruhi interaksi tersebut |  |  |  |  |  |
| 16.  During interactions with patients, I recognize when my patient’s pre-existing beliefs are influencing the interaction | 4.07 | 1.54 |  | 0.804 | 0.868 |
| 16. Selama berinteraksi dengan pasien, saya mengetahui ketika **keyakinan pasien** saya yang sudah ada sebelumnya memengaruhi interaksi tersebut |  |  |  |  |  |
| **Reflection on Action** | **4.16** | **1.01** | **0.87** |  | |
| 1.     After interacting with patients, I think about how things went during the interaction | 4.24 | 1.18 |  | 0.691 | 0.849 |
| 1. Setelah berinteraksi dengan pasien, saya memikirkan apa yang terjadi selama interaksi tersebut |  |  |  |  |  |
| 4.     After interacting with patients, I wonder about the patient’s experience of the interaction | 4.10 | 1.22 |  | 0.716 | 0.840 |
| 4. setelah berinteraksi dengan pasien, saya bertanya-tanya mengenai pengalaman pasien dari interaksi tersebut |  |  |  |  |  |
| 6.     After interacting with patients, I spend time thinking about what was said and done | 4.18 | 1.19 |  | 0.769 | 0.818 |
| 6. Setelah berinteraksi dengan pasien, saya meluangkan waktu untuk memikirkan apa yang telah diucapkan dan dilakukan |  |  |  |  |  |
| 8.     After interacting with patients, I wonder about my own experience of the interaction | 4.13 | 1.19 |  | 0.726 | 0.836 |
| 8. setelah berinteraksi dengan pasien, saya bertanya-tanya mengenai pengalaman saya dari interaksi tersebut |  |  |  |  |  |
| **Reflection with others** | **4.33** | **0.96** | **0.89** |  | |
| 2.     When reflecting with others about my work, I develop new perspectives | 4.39 | 1.10 |  | 0.786 | 0.832 |
| 1. Saat berefleksi dengan orang lain mengenai pekerjaan saya, saya memperoleh sudut pandang baru |  |  |  |  |  |
| 3.     I gain new insights when reflecting with others about my work | 4.47 | 1.11 |  | 0.790 | 0.830 |
| 3.saya mendapatkan wawasan baru ketika berefleksi dengan orang lain mengenai pekerjaan saya |  |  |  |  |  |
| 5.     I find that reflecting with others about my work helps me to work out problems I might be having | 4.21 | 1.15 |  | 0.717 | 0.858 |
| 5. Saya mendapati bahwa berefleksi dengan orang lain terkait pekerjaan saya, dapat membantu saya menyelesaikan masalah yang mungkin saya alami |  |  |  |  |  |
| 14.  When reflecting with others about my work, I become aware of things I had not previously considered | 4.25 | 1.12 |  | 0.683 | 0.871 |
| 14. ketika berefleksi dengan orang lain mengenai pekerjaan saya, saya menjadi sadar tentang hal-hal yang sebelumnya tidak saya pertimbangkan |  |  |  |  |  |
| **Self Appraisal** | **4.36** | **0.98** | **0.89** |  | |
| 7.     I think about my weaknesses for working with patients | 4.37 | 1.16 |  | 0.690 | 0.878 |
| 7. saya memikirkan tentang kelemahan saya dalam menangani pasien |  |  |  |  |  |
| 10.  I think about how I might improve my ability to work with patients | 4.57 | 1.14 |  | 0.776 | 0.845 |
| 10. Saya memikirkan cara meningkatkan kemampuan saya dalam menangani pasien |  |  |  |  |  |
| 12.  I critically evaluate the strategies and techniques I use in my work with patients | 4.24 | 1.13 |  | 0.761 | 0.851 |
| 12. Saya secara kritis mengevaluasi strategi dan teknik yang saya gunakan dalam menangani pasien. |  |  |  |  |  |
| 13.  I think about my strengths for working with patients | 4.28 | 1.13 |  | 0.785 | 0.842 |
| 13. saya memikirkan tentang kekuatan saya dalam menangani pasien |  |  |  |  |  |
| **Main scale – RPQ** | **4.25** | **0.91** | **0.96** |  | |

SD = standard deviation

CITD= corrected item-total correlation

AIID= alpha if item deleted

Figure 1. Reflective practice questionnaire (RPQ) with standardized path coefficient

Table 2. Goodness-of-fit RPQ

| **Fit Measure** | **Model Fit** | **Acceptable fit** |  |
| --- | --- | --- | --- |
| CFI (Comparative Fit Index) | 0.936 | > 0.90 |  |
| TLI (Tucker-Lewis Index) | 0.921 | > 0.90 |  |
| RMSEA (Root Mean Square Error of Approximation) | 0.093 | < 0.08 |  |
| SRMR (Standardized Root Mean Square Residual) | 0.041 | < 0.08 |  |

**1.2.2 SRIS psychometric analysis results**

We applied the translated version of SRIS to 1,522 participants, including 429 male and 1,093 female students, with a mean age of 20.98 years (SD = 2.16).

We performed a confirmatory factor analysis (CFA) of the original model. The results showed that it was possible to distinguish the three subscales. However, the model did not achieve an adequate fit because some items were not fitting well in the model. Therefore, to improve the goodness-of-fit indices, we removed seven items, i.e. three from the Engagement in Self-Reflection Reflection subscale, one from the Need for Self-Reflection subscale, and three from the Insight subscale (Table 3, Figure 2). After these modifications, the revised model demonstrated better performance across nearly all fit indices: CFI = 0.928, TLI = 0.910, RMSEA = 0.073, and SRMS = 0.057 (Table 4). The reliability of the revised SRIS model was 0.70, while the reliability for its subscales was 0.66 (Engagement in self-reflection), 0.79 (Need for self-reflection), and 0.86 (Insight). The correlation of the original subscales and the new subscales were acceptable ranging from 0.80 – 0.94 (Table 5).

Table 3. Item response statistics of the 20-item self-reflection and insight scale (SRIS)

| **Item** | **Mean** | **SD** | **Cronbach’s α (original)** | **CITC (original)** | **AIID (original)** | **Cronbach’s α (Revised)** | **CITC (Revised)** | **AIID (Revised)** |
| --- | --- | --- | --- | --- | --- | --- | --- | --- |
| **Engagement in self-reflection** | **3.37** | **0.52** | **0.52** |  | | **0.66** |  | |
| 1.     I don’t often think about my thoughts (R) | 3.15 | 1.13 |  | 0.303 | 0.454 |  | 0.432 | 0.617 |
| 1. Saya tidak sering memikirkan tentang pikiran saya (R) |  |  |  |  |  |  |  |  |
| 8.    I rarely spend time in self-reflection (R) | 2.87 | 1.05 |  | 0.401 | 0.394 |  | 0.469 | 0.565 |
| 8. Saya jarang meluangkan waktu untuk refleksi diri (R) |  |  |  |  |  |  |  |  |
| 10.  I frequently examine my feelings | 3.70 | 0.83 |  | 0.174 | 0.513 |  | Removed | Removed |
| 10. Saya sering memeriksa perasaan saya |  |  |  |  |  |  |  |  |
| 13.  I don’t really think about why I behave in the way that I do (R) | 3.05 | 1.06 |  | 0.256 | 0.479 |  | 0.512 | 0.506 |
| 13. Saya tidak terlalu memikirkan mengapa saya berperilaku seperti yang saya lakukan (R) |  |  |  |  |  |  |  |  |
| 16.  I frequently take time to reflect on my thoughts | 3.70 | 0.81 |  | 0.293 | 0.462 |  | Removed | Removed |
| 16. Saya sering meluangkan waktu untuk merefleksikan pikiran saya |  |  |  |  |  |  |  |  |
| 19.  I often think about the way I feel about things | 3.83 | 0.74 |  | 0.184 | 0.507 |  | Removed | Removed |
| 19. Saya sering memikirkan mengenai cara saya merasakan berbagai hal |  |  |  |  |  |  |  |  |
| **Need for self-reflection** | **3.89** | **0.70** | **0.70** |  | | **0.79** |  | |
| 2.     I am not really interested in analysing my behaviour (R) | 3.43 | 1.02 |  | 0.079 | 0.790 |  | Removed | Removed |
| 2. Saya tidak terlalu tertarik menelaah perilaku saya (R) |  |  |  |  |  |  |  |  |
| 5.     It is important for me to evaluate the things that I do | 4.15 | 0.70 |  | 0.506 | 0.626 |  | 0.510 | 0.768 |
| 5. Penting bagi saya untuk mengevaluasi hal yang saya lakukan |  |  |  |  |  |  |  |  |
| 7.     I am very interested in examining what I think about | 3.97 | 0.71 |  | 0.532 | 0.618 |  | 0.555 | 0.755 |
| 7. Saya sangat tertarik memeriksa apa yang saya pikirkan |  |  |  |  |  |  |  |  |
| 12.  It is important to me to try to understand what my feelings mean | 4.07 | 0.71 |  | 0.593 | 0.599 |  | 0.624 | 0.733 |
| 12. Penting bagi saya untuk bisa memahami makna perasaan saya |  |  |  |  |  |  |  |  |
| 15.  I have a definite need to understand the way my mind works | 3.94 | 0.78 |  | 0.515 | 0.619 |  | 0.583 | 0.746 |
| 15. Saya merasa butuh untuk memahami cara kerja pikiran saya |  |  |  |  |  |  |  |  |
| 18.  It is important to me to be able to understand how my thoughts arise | 3.81 | 0.78 |  | 0.488 | 0.628 |  | 0.572 | 0.749 |
| 2. Penting bagi saya untuk bisa mengerti cara munculnya pikiran saya |  |  |  |  |  |  |  |  |
| **Insight** | **3.05** | **0.54** | **0.75** |  | | **0.86** |  | |
| 3.     I am usually aware of my thoughts | 4.08 | 0.63 |  | 0.075 | 0.768 |  | Removed | Removed |
| 3. Saya biasanya sadar akan pikiran saya |  |  |  |  |  |  |  |  |
| 4.     I am often confused about the way that I really feel about things (R) | 2.35 | 0.97 |  | 0.551 | 0.696 |  | 0.635 | 0.845 |
| 4. Saya sering bingung mengenai apa sebenarnya yang saya rasakan tentang berbagai hal (R) |  |  |  |  |  |  |  |  |
| 6.     I usually have a very clear idea about why I have behaved in a certain way | 3.84 | 0.75 |  | 0.154 | 0.763 |  | Removed | Removed |
| 6. Saya biasanya mengetahui dengan jelas kenapa saya berperilaku tertentu |  |  |  |  |  |  |  |  |
| 9.     I’m often aware that I am having a feeling, but I often don’t quite know what it is (R) | 2.40 | 0.94 |  | 0.557 | 0.695 |  | 0.651 | 0.842 |
| 9. Saya sering menyadari saat saya merasakan sesuatu, tapi saya sering tidak mengetahui perasaan apa itu. (R) |  |  |  |  |  |  |  |  |
| 11.  My behaviour often puzzles me (R) | 2.71 | 1.07 |  | 0.658 | 0.669 |  | 0.726 | 0.823 |
| 11. Perilaku saya sering membuat saya bingung (R) |  |  |  |  |  |  |  |  |
| 14.  Thinking about my thoughts makes me more confused (R) | 2.68 | 1.02 |  | 0.617 | 0.680 |  | 0.726 | 0.823 |
| 14. Memikirkan tentang pikiran saya membuat saya semakin bingung (R) |  |  |  |  |  |  |  |  |
| 17.  Often I find it difficult to make sense of the way I feel about things (R) | 2.64 | 1.00 |  | 0.638 | 0.676 |  | 0.687 | 0.833 |
| 17. Saya sering merasa kesulitan memahami cara saya merasakan sesuatu (R) |  |  |  |  |  |  |  |  |
| 20.  I usually know why I feel the way I do | 3.74 | 0.78 |  | 0.168 | 0.762 |  | Removed | Removed |
| 20. Saya biasanya mengetahui kenapa saya merasakan perasaan tertentu |  |  |  |  |  |  |  |  |
| **SRIS – main scale** | 3.41 | 0.37 | **0.76** |  | | **0.70** |  | |

SD = standard deviation

CITD= corrected item-total correlation

AIID= alpha if item deleted

Figure 2. Self-reflection and insight scale (SRIS) with standardized path coefficient of the model fit

Table 4. Goodness-of-fit SRIS

| **Fit Measure** | **Original model** | **Revised Model** | **Acceptable fit** |
| --- | --- | --- | --- |
| CFI (Comparative Fit Index) | 0.539 | 0.928 | > 0.90 |
| TLI (Tucker-Lewis Index) | 0.475 | 0.910 | > 0.90 |
| RMSEA (Root Mean Square Error of Approximation) | 0.149 | 0.073 | < 0.08 |
| SRMR (Standardized Root Mean Square Residual) | 0.192 | 0.057 | < 0.08 |

Table 5. Correlation of original and revised model of SRIS

|  | **Engagement in self-reflection Original** | **Need for self-reflection Revised** | **Insight Original** | **SRIS Original** |
| --- | --- | --- | --- | --- |
| **Engagement in self-reflection Revised** | 0.80^**^ | 0.21^**^ | 0.42^**^ | 0.65^**^ |
| **Need for self-reflection Revised** | 0.38^**^ | 0.94^**^ | -0.11^**^ | 0.47^**^ |
| **Insight Revised** | 0.25^**^ | -0.21^**^ | 0.92^**^ | 0.55^**^ |
| **SRIS Revised** | 0.72^**^ | 0.39^**^ | 0.80^**^ | 0.91^**^ |

*. Correlation is significant at the 0.05 level (2-tailed).

**. Correlation is significant at the 0.01 level (2-tailed).

2. **Professional Identity Formation Questionnaire** ^6^

**Instructions:** Below are statements that indicate the perceptions or feelings of medical students towards the medical profession. Please place a check mark (√) in the answer column on the value that corresponds to how you feel at this moment. The scale ranges from 1 to 7, with 1 indicating the statement is very inappropriate for you, increasing up to 7 indicating the statement is very appropriate for you. Each number is also given the option N/A (not applicable), only if you have never experienced it at all.

Subscale Comfort

1. I feel that I belong to the medical professional community.
2. I am comfortable being part of the medical professional community.
3. I am pleased to be involved in patient management.
4. I am pleased to be trusted to perform medical procedures.
5. I would like to always be involved in patient management.

Subscale Enthusiasm

6. I enjoy participating in patient case discussions.
7. I am enthusiastic about answering medical questions.
8. I am enthusiastic about sharing my opinions during patient case discussions.
9. I strive to update my clinical skills according to scientific developments.
10. I am enthusiastic about participating in health-related programs.

Subscale Efficacy

11. CURRENTLY, if were asked to serve as a doctor, I would feel confident and ready.
12. CURRENTLY, if faced with a patient, I know exactly what steps to take.
13. CURRENTLY, I feel that my opinions have contributed to patient care.
14. CURRENTLY, I am capable of providing health education to patients or the community.
15. CURRENTLY, if assigned to work as a doctor in various settings (community health center, emergency room, outpatient or inpatient units), I am confident in my ability.
16. CURRENTLY, I feel that I have an important role in the healthcare team.
17. I am confident that my current abilities have contributed to the improvement of public health

Table 6. Comparison of main variables between school and cohorts

| **Variables** | | **PIF** | | | | |  | **RPQ** | | | | |  | **SRIS** | | | | |
| --- | --- | --- | --- | --- | --- | --- | --- | --- | --- | --- | --- | --- | --- | --- | --- | --- | --- | --- |
|  |  | **Mean** | **SD** | **Sig.** | **η^2^** | **ω^2^**  **random-effect** |  | **Mean** | **SD** | **Sig.** | **η^2^** | **ω^2^**  **random-effect** |  | **Mean** | **SD** | **Sig.** | **η^2^** | **ω^2^**  **random-effect** |
| School | 1 | 5.14 | 1.43 | 0.009 | 0.008 | 0.008 |  | 4.33 | 0.81 | 0.005 | 0.009 | 0.002 |  | 3.36 | 0.38 | 0.018 | 0.007 | 0.002 |
|  | 2 | 4.95 | 1.53 |  |  |  |  | 3.98 | 1.03 |  |  |  |  | 3.41 | 0.38 |  |  |  |
|  | 3 | 4.74 | 1.77 |  |  |  |  | 4.25 | 0.93 |  |  |  |  | 3.46 | 0.37 |  |  |  |
|  | 4 | 5.11 | 1.67 |  |  |  |  | 4.25 | 0.97 |  |  |  |  | 3.40 | 0.37 |  |  |  |
| Academic year | Year 1  pre-clinical | 4.45 | 1.87 | <0.001 | 0.104 | 0.022 |  | 4.13 | 0.93 | <0.001 | 0.024 | 0.004 |  | 3.19 | 0.43 | 0.136 | 0.006 | 0.000 |
|  | Year 2  pre-clinical | 4.51 | 1.77 |  |  |  |  | 4.11 | 0.94 |  |  |  |  | 3.13 | 0.39 |  |  |  |
|  | Year 3  pre-clinical | 4.80 | 1.65 |  |  |  |  | 4.14 | 0.99 |  |  |  |  | 3.22 | 0.41 |  |  |  |
|  | Year 4  pre-clinical | 4.89 | 1.79 |  |  |  |  | 4.27 | 1.01 |  |  |  |  | 3.25 | 0.45 |  |  |  |
|  | Year 1 clinical | 5.65 | 1.00 |  |  |  |  | 4.49 | 0.77 |  |  |  |  | 3.21 | 0.45 |  |  |  |
|  | Year 2 clinical | 5.78 | 0.90 |  |  |  |  | 4.36 | 0.82 |  |  |  |  | 3.23 | 0.45 |  |  |  |

**References**

1.	Beaton DE, Bombardier C, Guillemin F, Ferraz MB. Guidelines for the process of cross-cultural adaptation of self-report measures. *Spine*. 2000;25(24):3186–3191. https://doi.org/10.1097/00007632-200012150-00014

2.	Tsang S, Royse C, Terkawi A. Guidelines for developing, translating, and validating a questionnaire in perioperative and pain medicine. *Saudi J Anaesth*. 2017;11(5):80. https://doi.org/10.4103/sja.sja_203_17

3.	MacCallum RC, Browne MW, Sugawara HM. Power Analysis and Determination of Sample Size for Covariance Structure Modeling. *Psychological Methods*. 1996;1(2):130–49.

4.	Hu L, Bentler PM. Cutoff criteria for fit indexes in covariance structure analysis: Conventional criteria versus new alternatives. *Structural Equation Modeling: A Multidisciplinary Journal*. 1999;6(1):1–55. https://doi.org/10.1080/10705519909540118

5.	Nunally JC, Bernstein IH. The Assessment of Reliability. *Psychometric Theory*. Vol. 3. 1994:248–92.

6.	Susani YP, Rahayu GR, Sanusi R, Prabandari YS, Mardiwiyoto H. Developing a model of professional identity in medical students: The role of motivation and participation. *JPKI*. 2018;7(3):159. https://doi.org/10.22146/jpki.41831
